# Supplementary material for: Opinion manipulation on Farsi Twitter
Source: Sci Rep. 2023 Jan 6;13:333. doi: 10.1038/s41598-022-26921-5 (PMC9823014; doi:10.1038/s41598-022-26921-5)
Supplement: Supplementary file 1 — Supplementary Information. [file 41598_2022_26921_MOESM1_ESM.pdf]

# Supplementary Information for Opinion Manipulation on Farsi Twitter

**Amirhossein Farzam[1†,2,3, *], Parham Moradi[4,5], Saeedeh Mohammadi[4,5, 6†], Zahra Padar[7], and Alexandra A. Siegel[8, *]**

[1]Department of Political Science, Duke University, Durham, NC 27708
[2]Department of Engineering Sciences and Applied Mathematics, Northwestern University, Evanston, IL 60208
[3]Max Planck Institute for Mathematics in the Sciences, 04103 Leipzig, Germany
[4]Center for Complex Networks and Social Data Science, Tehran, Iran
[5]Department of Physics, Shahid Beheshti University, Tehran, Iran
[6]School of Mathematics and Statistics, University College Dublin, Belfield, Dublin 4, Ireland
[7]Department of Computer Science, University of Freiburg, Freiburg, Germany
[8]Department of Political Science, University of Colorado, Boulder, CO 80309

## Other Discussion Topics

Throughout the Results section in the main body of this paper, we include figures and discuss results comparing divisive political discussions against apolitical discussions. In the cases where the analysis is performed for each discussion separately, we include the results for on two discussion topics: Snapp (divisive political) and Valentine (apolitical). These two topics are included as examples in the main manuscript. In this appendix, we include figures for the results, which extend to other topics within each discussion type. Additionally, we also include distributions of complete automation probability (CAP) across all types of discussions, visualizations of all networks, as well as an analysis of the similarity of the results from different runs of the Louvain clustering algorithm on each network.

### Distribution of Complete Automation Probabilities

We begin our Results section in the main manuscript with a discussion on prevalence of inauthentic activities across different discussion types. As we discuss in the manuscript, this paper primarily aims to study inauthentic activities in divisive political discussions compared against apolitical discussions which serve as a benchmark. The other category of discussion —non-divisive political discussions— provide a safety margin, separating the two types of discussions. To present an overall view of inauthentic activities in this category, in this appendix we include the distribution of Complete Automation Probabilities (CAP) in this type of discussions, for users belonging to the top decile of each of the three influence measures in the retweet network, as we discuss in the manuscript for apolitical and divisive political discussions. The distributions are shown in Fig. 1.

### Network Communities and Visualisation

In this section of the appendix, we include network visualizations for discussions in both target categories, with the nodes color coded by network community, in Figures 2 through 8. These visualizations show friendship and retweet networks, and for each, we include two visualizations with two different color codings: one reflecting the communities in the same network, and one reflecting the communities in the other network. That is, for each discussion, we include four visualizations: friendship network with nodes color coded by friendship communities, friendship network with nodes color coded by retweet communities, retweet network with nodes color coded by retweet communities, retweet network with nodes color coded by friendship communities. Please note that in these visualisations only 7 major communities of networks are demonstrated and smaller, isolated communities are removed to better represent the structure of the main network. Please further note that accounts that participate in the discussion, but neither retweeted a tweet nor were retweeted by other accounts, correspond to

* Corresponding authors: a.farzam@duke.edu, Alexandra.Siegel@colorado.edu
†Current affiliation
For a major part of this study, A.F. was affiliated with the Department of Engineering Sciences and Applied Mathematics at Northwestern University in Evanston, Illinois, and the Max Planck Institute for Mathematics in the Sciences in Leipzig, Germany, and S.M. was affiliated with the Center for Complex Networks and Social Data Science and the Department of Physics at Shahid Beheshti University in Tehran, Iran.

nodes in the friendship network that do not exist in the retweet network. These nodes are colored white in the visualizations of friendship networks reflecting retweet communities.

In the Results section we discuss the distribution of suspended and deactivated accounts in different retweet network communities for divisive political discussions compared against apolitical discussions, and also compared the retweet and friendship network communities. In particular, we discuss the similarities between retweet and friendship communities in the Inauthentic Activity in Echo Chambers subsection of the main manuscript, where we include visualizations of the network adjacency matrices for the Snapp (divisive political) and Valentine (apolitical) discussions with rows and columns permuted such that nodes belonging to the same community are grouped together. These sparsity patterns of the adjacency matrices are demonstrated in Fig. 11 in this appendix.

As we discuss in the Results as well as the Methods sections of the main manuscript, we use the Louvain clustering algorithm to detect network communities in our data. In addition to considering the adjacency matrix structure shown in Fig. 11, to further confirm the reliability of our community detection for our macro-scale analysis, we construct an ensemble of 100 clusterings obtained from different runs of the Louvain algorithm. We then compute the pairwise similarities between the network clustering we use in other analyses and each of the alternative clusterings, with respect to two similarity measures —Jaccard index and element-centric similarity—, as we explain in the Methods section of the main manuscript. The results, included in Figures 9 and 10, indicate that the vast majority of similarity values fall above 0.8 (out of a maximum similarity of 1) with nearly all of the values above 0.5, which confirms that our observations and interpretations are not sensitive to differences between different runs of the algorithm, considering that our analyses look at the network at the macro scale and draw aggregate-level conclusions.

## Participation in Trending Discussions

We show the differences between the Snapp (divisive political) and Valentine (apolitical) discussions with respect to the temporal pattern of participation by different groups of users in the Results section of the main manuscript. As it is shown by Spearman correlations visualized in Fig. 12, the discussion in the main manuscript generally holds across other discussions of the same types with the exception of the apolitical discussion on Depression, which deviates from this general pattern. The correlation difference between groups A and C in the Trump discussion is also not as pronounced as it is in the Blue Girl and Snapp discussions, but this is in line with our general understanding about the more mixed collection of tweets on the Trump discussion, as opposed to the more unequivocally divisive discussions about Blue Girl and Snapp.

## Differences in Content

Consistent with other results reported, for the differences in the content of tweets posted by users from different user types and network communities, in the Results section of the main manuscript we report the results for the Snapp and Valentine discussions as representatives of divisive political and apolitical discussions. In this part of this appendix, we include the word clouds as well as detected topics with their representative words for other divisive political and apolitical discussions, constructed as described in the Results and Methods sections of the main manuscript. The observations in Figures 13 through 21 confirm that the content analysis discussed in the main manuscript can be generally extended to other discussions in the two target categories. Note that the word clouds for three apolitical discussions were empty showing no difference between the two communities, and hence were not included in Fig. 13.

## Topics of Discussion

In this appendix we explain the topics of discussion we chose for this paper. We analyzed the discussion space of the following eleven topics:

- **Bahareh Hedayat:** This discussion started after the arrest of Bahareh Hedayat - a student activist at the University of Tehran. The discussion was limited in scale as the news did not find its way to mainstream media. Nevertheless, Twitter accounts from both sides of the political spectrum tweeted about it. The topic is political, but the dominant voice belonged to groups opposing the arrest - including some Iranian officials affiliated with the government or journalists active in Iran. Therefore, this is a non-divisive political discussion. Our data for this discussion contains 9794 tweets from 4651 unique user IDs.
- **Blue Girl:** This discussion took place after a female soccer fan in Iran committed political self-immolation in public in protest to the existing ban on women entering soccer stadiums. The discussion was one of the main trending discussions at the time, and users from across the political spectrum tweeted about the issue. Since the more conservative groups in Iran believe that the ban is in the interest of the public, the discussion space was quite polarized. This discussion is a divisive political discussion. Our data for this discussion contains 539682 tweets from 57616 unique user IDs.
- **Depression:** This topic is endemic to Twitter. A user posted her selfie with the caption *"This is the face of depression"*. Since the user—a woman— was wearing revealing clothes in the picture, it was deemed provocative and the intention of the user was questioned by Farsi Twitter users. This was followed by both a series of parody selfies and a series of serious tweets on the issue of depression. This discussion remained mainly social and apolitical. Our data for this discussion contains 5016 tweets from 3943 unique user IDs.
- **Drone:** This discussion followed the downing of the American drone in the Persian Gulf in 2019. The event was a part of a sequence of events heightening the tensions between the governments of Iran and the United States. Many Iranians were genuinely concerned about a seemingly imminent conflict. The discussion space was dominated by the supporters of the Iranian government praising the Iranian military for its response. Many dissident groups did not participate in this discussion and, although political, the discussion was mostly non-divisive. Our data for this discussion contains 40201 tweets from 15916 unique user IDs.
- **Golab Adineh:** This discussion started after the news about a controversial celebrity marriage, following a celebrity divorce, broke out. Mehdi Hashemi, a renowned Iranian actor, then 72 years old, married Mehnoosh Sadeghi, an actress who was at the time 46 years old. Mehdi Hashemi was previously married to Golab Adineh, a then 65 year old famous actress, who was separated from Mehdi Hashemi for a while before their recent divorce. The discussion sparked a social controversy about second marriage and women's rights. Although it was a heated-up social discussion, it never became political. Our data for this discussion contains 5962 tweets from 4117 unique user IDs.
- **Kobe Bryant:** A series of tweets from a variety of accounts followed the death of the NBA athlete, Kobe Bryant. Although similar attentions to such topics linked to western culture is typically potent of sparking controversy, with possible ideological or political flavor, this discussion remained apolitical. Our data for this discussion contains 3662 tweets from 2872 unique user IDs.
- **Plane:** Following the downing of the Ukrainian plane in Iran, which itself followed the assassination of Qasem Soleimani, Farsi Twitter became a vibrant scene of political discussion about the incident. Since no group or individual approved of the downing of the plane, users on one end of the political spectrum remained mostly silent and the discussion space was dominated by those opposing the Iranian government and condemning the action. Although in this respect this discussion shares characteristics with non-divisive political discussions, given the unique context with highly divisive prelude and consequences, this discussion stands out as an isolated case per se. Our data for this discussion contains 123614 tweets from 37552 unique user IDs.
- **Snapp:** A driver of the most popular ride-sharing app in Iran, Snapp, asked his passenger to cover her hair with her headscarf in observation of the existing Hijab laws in Iran making wearing a headscarf mandatory for women. Following the passenger's refusal to comply, the driver terminated the ride and asked her to leave the car. The passenger posted her experience on social media and publicly asked the ride-sharing company, Snapp, to be responsible for their driver's behavior. The company initially sided with the customer, but following a series of tweets by the more conservative groups supporting the driver, they retracted their initial statement and supported the driver. The state TV and a number government officials spoke out about the incident supporting the driver's stance and praised him for trying to uphold the hijab law. This was followed by a reaction from the groups that oppose mandatory hijab, some of whom directly targeted

the Iranian government and pivoted into discussing more general issues about social freedom, women's rights, and the general approach of the Iranian government towards gender equality. The discussion became extremely polarized and remained a divisive political discussion. Our data for this discussion contains 144347 tweets from 33253 unique user IDs.

- **Suicide:** Saeed Namaki, the then Iranian Minster of Health and a relatively progressive figure in the Iranian political scene, made a comment about suicide and depression in Iran. This coincided with a recent suicide that made its way to the news and triggered a discussion on Twitter. Since a political figure was involved, some users linked depression in Iran to political issues and the discussion became political, while being simultaneously a social discussion. It was mainly non-divisive. Our data for this discussion contains 112783 tweets from 41665 unique user IDs.

- **Trump:** In the height of the tension between the governments of the United States and Iran in the spring and summer of 2019, following the attack on the oil tankers in the Persian Gulf, the Aramco attack, the downing of the American drone, and tightened sanctions, the US president *Donald Trump* became a trending topic of discussion in Iran. Although the related issues were less divisive, a considerable population of users in Farsi twitter who were against the Iranian government chose to support the stance of Trump's administration. On the other hand, the supporters of the Iranian government and a noticeable part of those opposing the government, were against Trump. Therefore, this discussion became a politically divisive one. Note that this discussion, unlike the others, had more than one peak. The data was collected over a long period and there were multiple peaks in the trendiness of the discussion. Our data for this discussion contains 112864 tweets from 29416 unique user IDs.

- **Valentine:** Before the Valentine's day in 2019, discussions about Valentine became trending in Farsi Twitter and remained trending until a few days after the Valentine's day. Since Valentine is not traditionally observed in Iran, it is considered an imported western tradition and the conservative groups of the society stand against its celebration. Meanwhile, it is widely observed by many groups of the urban population in Iran who support the celebration of this day. Despite its potentials for becoming political, this discussion remained apolitical. Our data for this discussion contains 7962 tweets from 5424 unique user IDs.

## List of Figures


1 The distributions of CAP scores among the most influential accounts, by PageRank (top), h-index (middle), and retweet count (bottom), in the retweet network of the Apolitical(left), Non-divisive Political (middle), and Divisive Political (right) discussions. The violin plots, and the quartile lines within each violin plot, show the distribution of CAP scores for users among the top decile of accounts with respect to the corresponding measure, in each topic within each category of discussion. . . . 7

2 The retweet and friendship networks for the Twitter discussion about Snapp. **Top left:** The friendship network color coded according the communities in the same friendship network. **Top right:** The retweet network color coded according to the communities in the same retweet network. **Bottom left:** The friendship network color coded according the communities in the corresponding retweet network. **Bottom right:** The retweet network color coded according to the communities in the corresponding friendship network. Excluding the isolated nodes, the retweet network for this discussion has 21029 nodes and 69565 edges, and the friendship network has 22993 nodes and 1728354 edges. Small clusters are excluded from this visualization. The retweet networks visualized in this figure contain 17483 nodes and 60768 edges, and the friendship networks contain 22991 nodes and 1728352 edges. Note that the grey dots, which are more abundant in the bottom row, mark those nodes that were not in any of the major communities. . . . 8

3 The retweet and friendship networks for the Twitter discussion about Blue Girl. **Top left:** The friendship network color coded according the communities in the same friendship network. **Top right:** The retweet network color coded according to the communities in the same retweet network. **Bottom left:** The friendship network color coded according the communities in the corresponding retweet network. **Bottom right:** The retweet network color coded according to the communities in the corresponding friendship network. Excluding the isolated nodes, the retweet network for this discussion has 47274 nodes and 320041 edges, and the friendship network has 15898 nodes and 1574559 edges. Small clusters are excluded from this visualization. The retweet networks visualized in this figure contain 45440 nodes and 315411 edges, and the friendship networks contain 15785 nodes and 1561803 edges. . . . 9

4 The retweet and friendship networks for the Twitter discussion about Trump. **Top left:** The friendship network color coded according the communities in the same friendship network. **Top right:** The retweet network color coded according to the communities in the same retweet network. **Bottom left:** The friendship network color coded according the communities in the corresponding retweet network. **Bottom right:** The retweet network color coded according to the communities in the corresponding friendship network. Excluding the isolated nodes, the retweet network for this discussion has 18596 nodes and 44254 edges, and the friendship network has 5372 nodes and 235668 edges. Small clusters are excluded from this visualization. The retweet networks visualized in this figure contain 15044 nodes and 39667 edges, and the friendship networks contain 5354 nodes and 234544 edges. . . . 10

5 The retweet and friendship networks for the Twitter discussion about Valentine. **Top left:** The friendship network color coded according the communities in the same friendship network. **Top right:** The retweet network color coded according to the communities in the same retweet network. **Bottom left:** The friendship network color coded according the communities in the corresponding retweet network. **Bottom right:** The retweet network color coded according to the communities in the corresponding friendship network. Excluding the isolated nodes, the retweet network for this discussion has 3705 nodes and 4491 edges, and the friendship network has 4001 nodes and 369965 edges. Small clusters are excluded from this visualization. The retweet networks visualized in this figure contain 1760 nodes and 2623 edges, and the friendship networks contain 3981 nodes and 369025 edges. . . . 11

6 The retweet and friendship networks for the Twitter discussion about Kobe Bryant. **Top left:** The friendship network color coded according the communities in the same friendship network. **Top right:** The retweet network color coded according to the communities in the same retweet network. **Bottom left:** The friendship network color coded according the communities in the corresponding retweet network. **Bottom right:** The retweet network color coded according to the communities in the corresponding friendship network. Excluding the isolated nodes, the retweet network for this discussion has 1783 nodes and 1748 edges, and the friendship network has 227 nodes and 749 edges. Small clusters are excluded from this visualization. The retweet networks visualized in this figure contain 1783 nodes and 1748 edges, and the friendship networks contain 211 nodes and 709 edges. . . . 12

7 The retweet and friendship networks for the Twitter discussion about Depression. **Top left:** The friendship network color coded according the communities in the same friendship network. **Top right:** The retweet network color coded according to the communities in the same retweet network. **Bottom left:** The friendship network color coded according the communities in the corresponding retweet network. **Bottom right:** The retweet network color coded according to the communities in the corresponding friendship network. Excluding the isolated nodes, the retweet network for this discussion has 2655 nodes and 2727 edges, and the friendship network has 1113 nodes and 25589 edges. Small clusters are excluded from this visualization. The retweet networks visualized in this figure contain 1115 nodes and 1230 edges, and the friendship networks contain 1113 nodes and 25589 edges. . . . 13

8 The retweet and friendship networks for the Twitter discussion about Golab Adineh. **Top left:** The friendship network color coded according the communities in the same friendship network. **Top right:** The retweet network color coded according to the communities in the same retweet network. **Bottom left:** The friendship network color coded according the communities in the corresponding retweet network. **Bottom right:** The retweet network color coded according to the communities in the corresponding friendship network. Excluding the isolated nodes, the retweet network for this discussion has 2118 nodes and 2192 edges, and the friendship network has 2676 nodes and 78339 edges. Small clusters are excluded from this visualization. The retweet networks visualized in this figure contain 910 nodes and 998 edges, and the friendship networks contain 2668 nodes and 78128 edges. . . . 14

9 The distribution of similarity values between the Louvain clustering used in our analysis for the friendship networks and 100 alternative clusterings of the same networks obtained from 100 runs of the algorithms using different random seeds. On the left, the similarities are computed according to element-centric similarity. On the right, the similarities are computed according to Jaccard index. . . . . . . . . 15
10 The distribution of similarity values between the Louvain clustering used in our analysis for the retweet networks and 100 alternative clusterings of the same networks obtained from 100 runs of the algorithms using different random seeds. On the left, the similarities are computed according to element-centric similarity. On the right, the similarities are computed according to Jaccard index. . . . . . . . . 16
11 The symmetrized retweet networks for each of the divisive political (top row) apolitical (the rest) Twitter discussions. In the upper triangle the rows and columns are reordered such to group nodes by their retweet communities. In the lower triangle, the reordering groups nodes by the community they belong to in the corresponding friendship network. Excluding the isolated nodes, the retweet network of the Snapp discussion has 21029 nodes and 69565 edges, and the retweet network of the Valentine discussion has 3705 nodes and 4491 edges. . . . . . 17
12 The Spearman correlation between the changes in the number of tweets from one day to the next for different groups of users — automated inauthentic accounts (group A), bot assisted humans and trolls (group B), and genuine users (group C). Please refer to Appendix section Topics of Discussion for descriptions of discussion topics and the number of tweets for each topic. . . . . . . . . . . . . . . . . . . 18
13 Difference word clouds by CAP for the Snapp, Blue Girl, Trump, and Valentine discussions, from top to bottom. Left: top 5% most frequent words used by users in major community 1 which are not among the top 10% most frequent words used by users in major community 2. Right: top 5% most frequent words used by users in major community 2 which are not among the top 10% most frequent words used by users in major community 1. Note that the difference word clouds for the apolitical discussions about Kobe Bryant, Depression, and Golab Adineh were empty, i.e. there is no difference between the two communities, in line with our main results. . . . . 19
14 Difference word clouds by CAP for the Snapp, Blue Girl, Trump, Valentine, Kobe Bryant, Depression, and Golab Adineh discussions, from top to bottom. Left: top 5% most frequent words across the high-CAP group which are absent from the top 10% most frequent words used by the low-CAP group. Right: top 5% most frequent words across the low-CAP group which are absent from the top 10% most frequent words used by the high-CAP group. . . . . . . . . . . . . . . . . . . . . . . . . . . . . . . . . . . . . . . . . . 20
15 Salient topics and representative words, obtained from BERTopic, for tweets from low-CAP (C) and high-CAP (A) on the top two rows and from user in two major friendship communities on the bottom two rows, for the Snapp discussion. . . . . . . . . . . . . . . . . 21
16 Salient topics and representative words, obtained from BERTopic, for tweets from low-CAP (C) and high-CAP (A) on the top two rows and from user in two major friendship communities on the bottom two rows, for the Blue Girl discussion. . . . . . . . . . . . . . . . 22
17 Salient topics and representative words, obtained from BERTopic, for tweets from low-CAP (C) and high-CAP (A) on the top two rows and from user in two major friendship communities on the bottom two rows, for the Trump discussion. . . . . . . . . . . . . . . . . 23
18 Salient topics and representative words, obtained from BERTopic, for tweets from low-CAP (C) and high-CAP (A) on the top two rows and from user in two major friendship communities on the bottom two rows, for the Valentine discussion. . . . . . . . . . . . . . . . 24
19 Salient topics and representative words, obtained from BERTopic, for tweets from low-CAP (C) and high-CAP (A) on the top two rows and from user in two major friendship communities on the bottom two rows, for the Kobe Bryant discussion. . . . . . . . . . . . . . . 25
20 Salient topics and representative words, obtained from BERTopic, for tweets from low-CAP (C) and high-CAP (A) on the top two rows and from user in two major friendship communities on the bottom two rows, for the Depression discussion. . . . . . . . . . . . . . . 26
21 Salient topics and representative words, obtained from BERTopic, for tweets from low-CAP (C) and high-CAP (A) on the top two rows and from user in two major friendship communities on the bottom two rows, for the Golab Adineh discussion. . . . . . . . . . . . . . 27

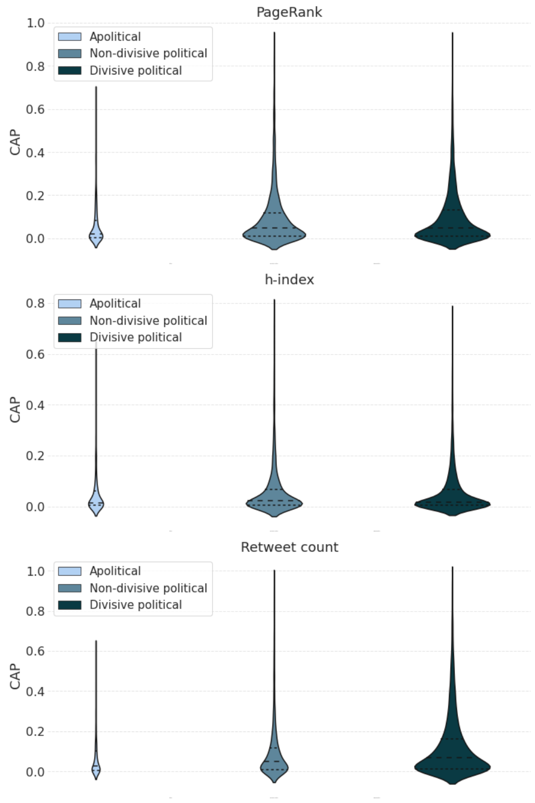


**Figure 1.** The distributions of CAP scores among the most influential accounts, by PageRank (top), h-index (middle), and retweet count (bottom), in the retweet network of the Apolitical(left), Non-divisive Political (middle), and Divisive Political (right) discussions. The violin plots, and the quartile lines within each violin plot, show the distribution of CAP scores for users among the top decile of accounts with respect to the corresponding measure, in each topic within each category of discussion.

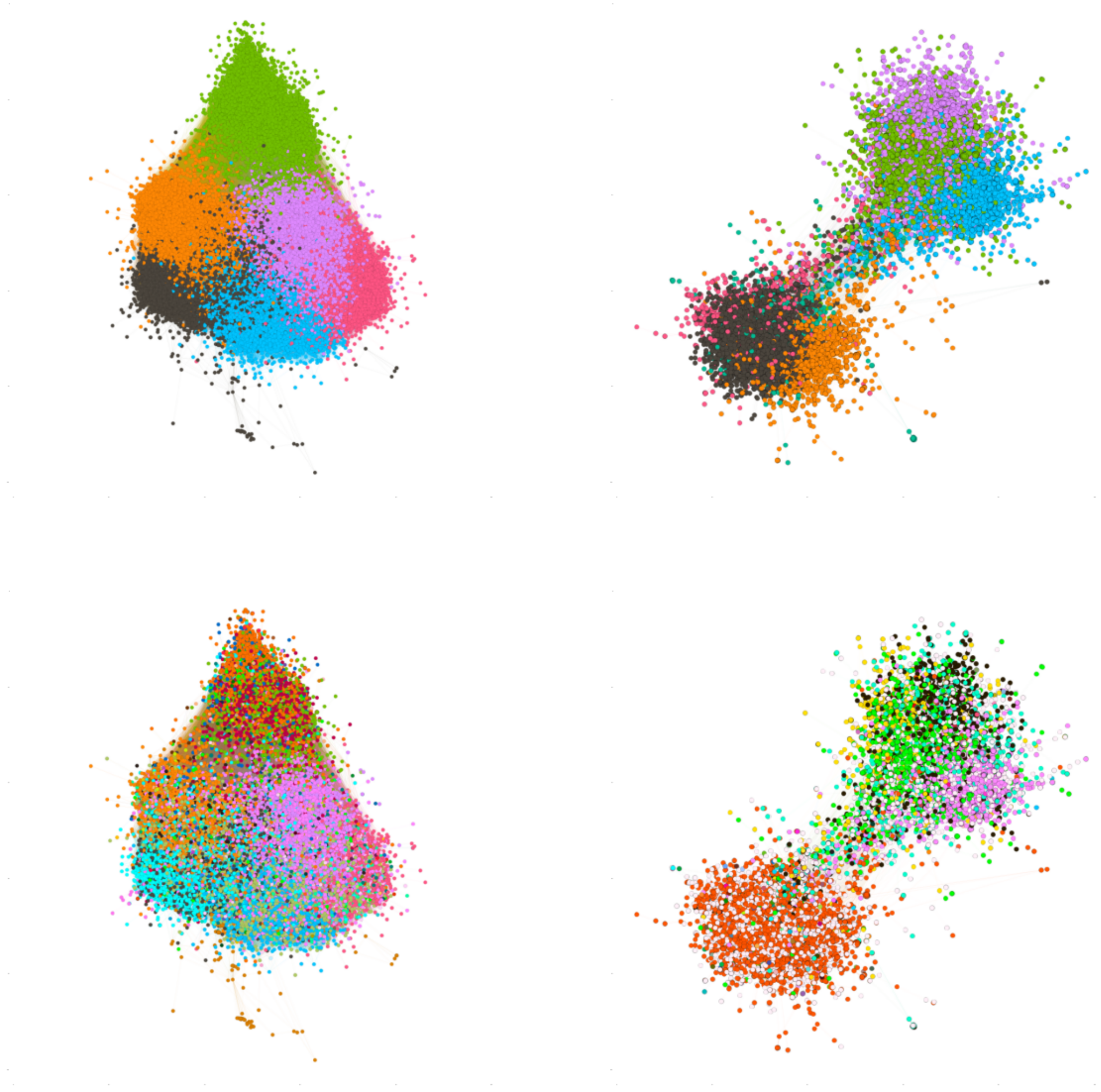

**Figure 2.** The retweet and friendship networks for the Twitter discussion about Snapp. **Top left:** The friendship network color coded according the communities in the same friendship network. **Top right:** The retweet network color coded according to the communities in the same retweet network. **Bottom left:** The friendship network color coded according the communities in the corresponding retweet network. **Bottom right:** The retweet network color coded according to the communities in the corresponding friendship network. Excluding the isolated nodes, the retweet network for this discussion has 21029 nodes and 69565 edges, and the friendship network has 22993 nodes and 1728354 edges. Small clusters are excluded from this visualization. The retweet networks visualized in this figure contain 17483 nodes and 60768 edges, and the friendship networks contain 22991 nodes and 1728352 edges. Note that the grey dots, which are more abundant in the bottom row, mark those nodes that were not in any of the major communities.

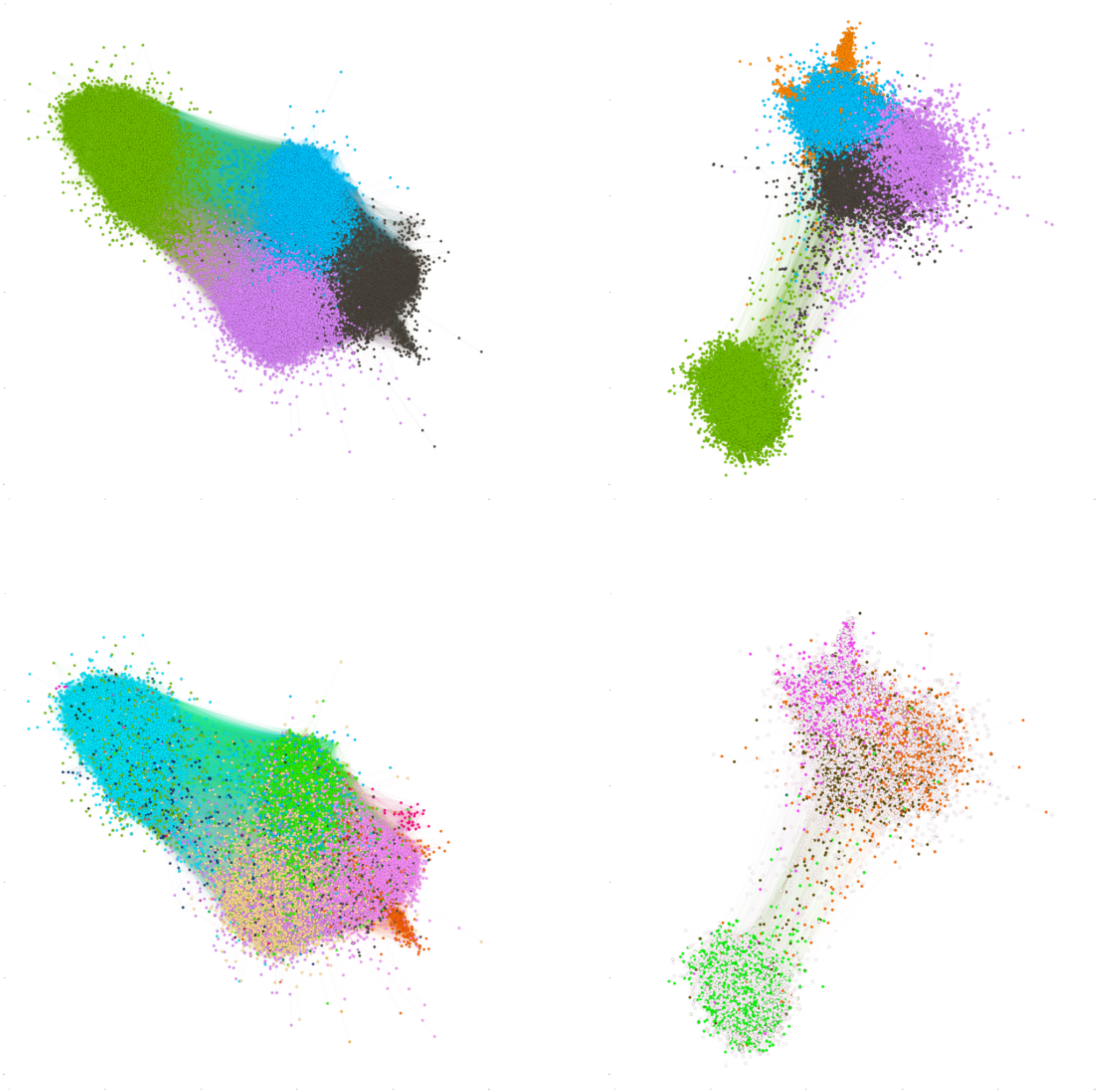

**Figure 3.** The retweet and friendship networks for the Twitter discussion about Blue Girl. **Top left:** The friendship network color coded according the communities in the same friendship network. **Top right:** The retweet network color coded according to the communities in the same retweet network. **Bottom left:** The friendship network color coded according the communities in the corresponding retweet network. **Bottom right:** The retweet network color coded according to the communities in the corresponding friendship network. Excluding the isolated nodes, the retweet network for this discussion has 47274 nodes and 320041 edges, and the friendship network has 15898 nodes and 1574559 edges. Small clusters are excluded from this visualization. The retweet networks visualized in this figure contain 45440 nodes and 315411 edges, and the friendship networks contain 15785 nodes and 1561803 edges.

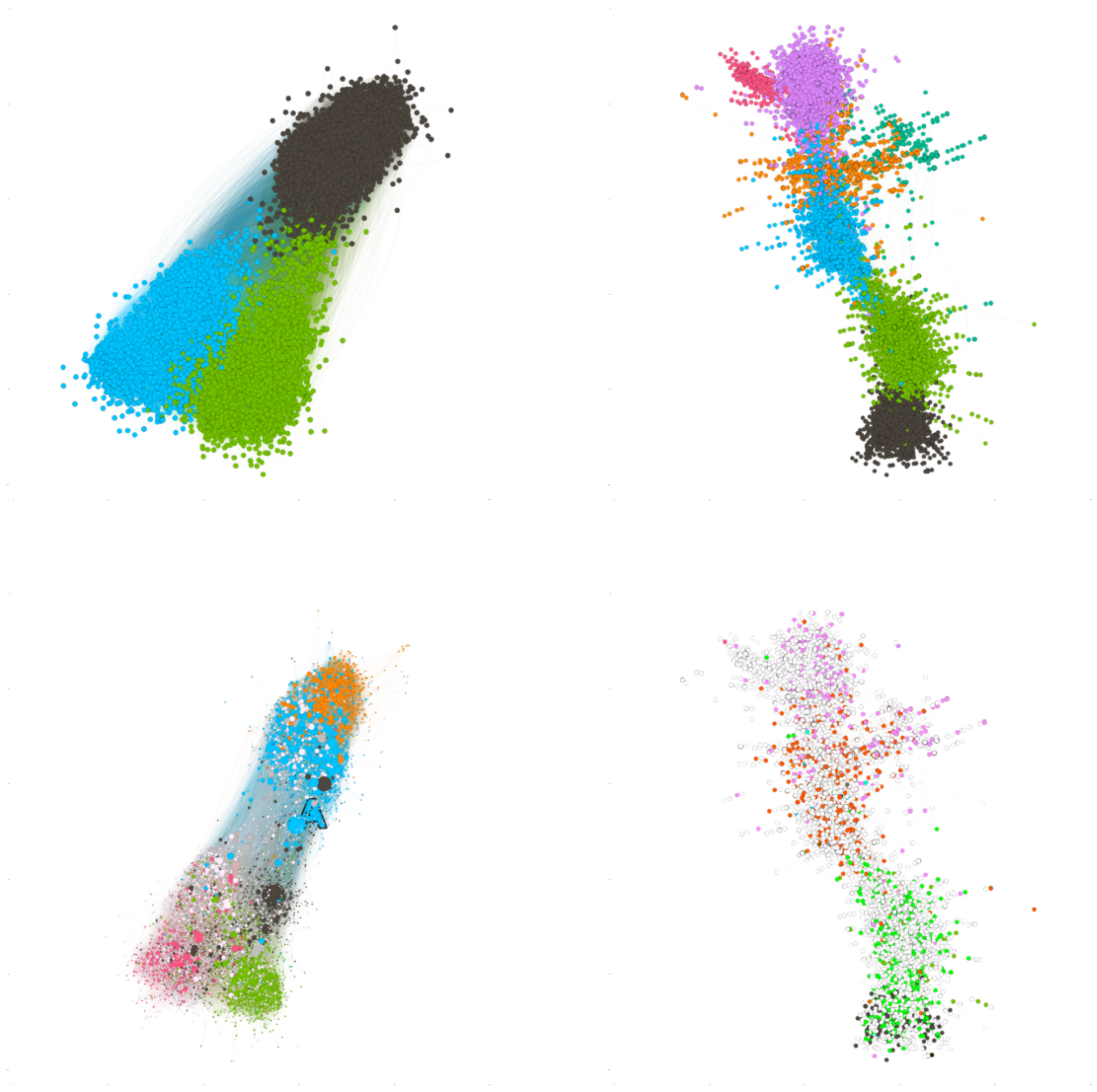

**Figure 4.** The retweet and friendship networks for the Twitter discussion about Trump. **Top left:** The friendship network color coded according the communities in the same friendship network. **Top right:** The retweet network color coded according to the communities in the same retweet network. **Bottom left:** The friendship network color coded according the communities in the corresponding retweet network. **Bottom right:** The retweet network color coded according to the communities in the corresponding friendship network. Excluding the isolated nodes, the retweet network for this discussion has 18596 nodes and 44254 edges, and the friendship network has 5372 nodes and 235668 edges. Small clusters are excluded from this visualization. The retweet networks visualized in this figure contain 15044 nodes and 39667 edges, and the friendship networks contain 5354 nodes and 234544 edges.

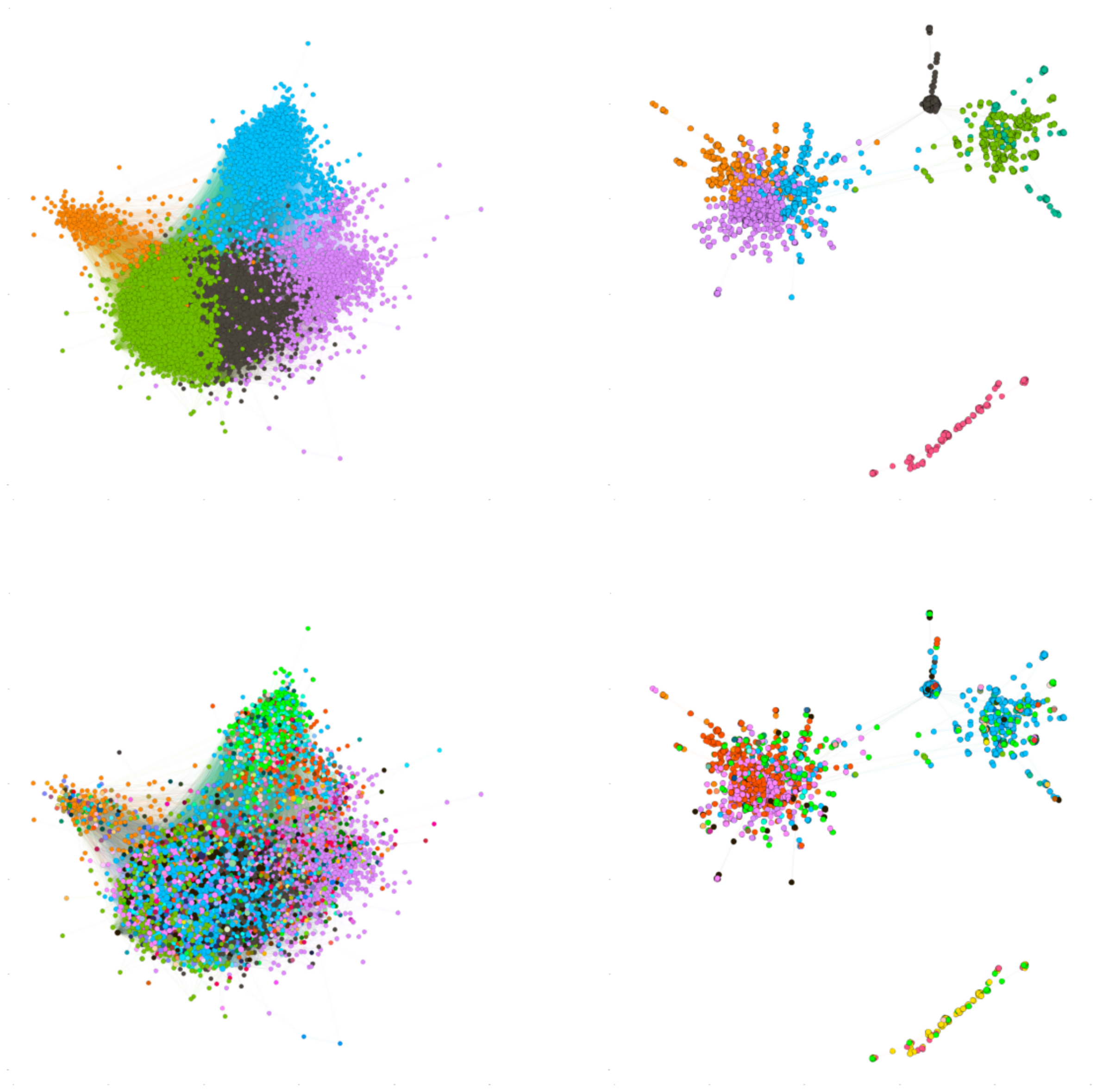

**Figure 5.** The retweet and friendship networks for the Twitter discussion about Valentine. **Top left:** The friendship network color coded according the communities in the same friendship network. **Top right:** The retweet network color coded according to the communities in the same retweet network. **Bottom left:** The friendship network color coded according the communities in the corresponding retweet network. **Bottom right:** The retweet network color coded according to the communities in the corresponding friendship network. Excluding the isolated nodes, the retweet network for this discussion has 3705 nodes and 4491 edges, and the friendship network has 4001 nodes and 369965 edges. Small clusters are excluded from this visualization. The retweet networks visualized in this figure contain 1760 nodes and 2623 edges, and the friendship networks contain 3981 nodes and 369025 edges.

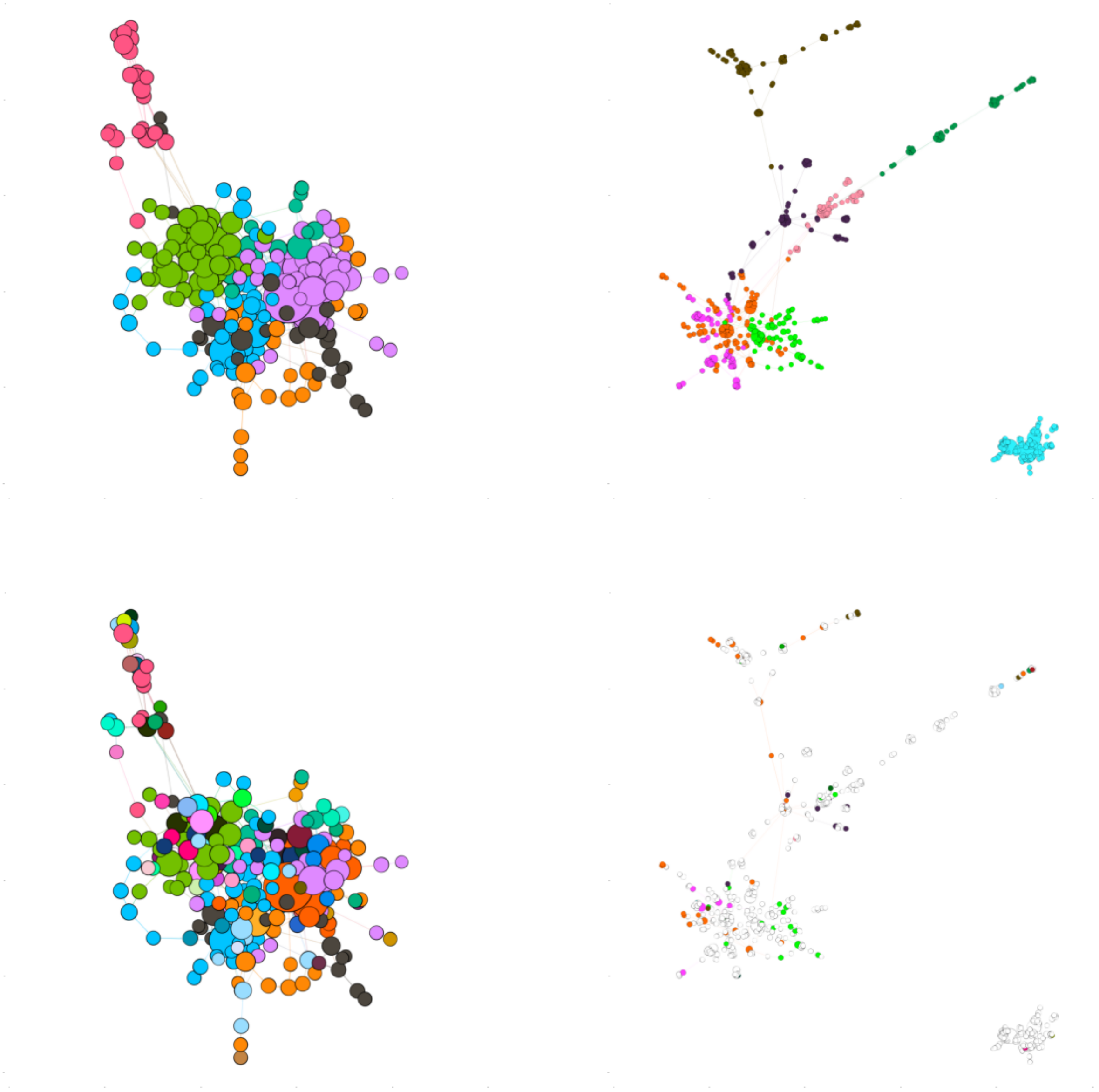

**Figure 6.** The retweet and friendship networks for the Twitter discussion about Kobe Bryant. **Top left:** The friendship network color coded according the communities in the same friendship network. **Top right:** The retweet network color coded according to the communities in the same retweet network. **Bottom left:** The friendship network color coded according the communities in the corresponding retweet network. **Bottom right:** The retweet network color coded according to the communities in the corresponding friendship network. Excluding the isolated nodes, the retweet network for this discussion has 1783 nodes and 1748 edges, and the friendship network has 227 nodes and 749 edges. Small clusters are excluded from this visualization. The retweet networks visualized in this figure contain 1783 nodes and 1748 edges, and the friendship networks contain 211 nodes and 709 edges.

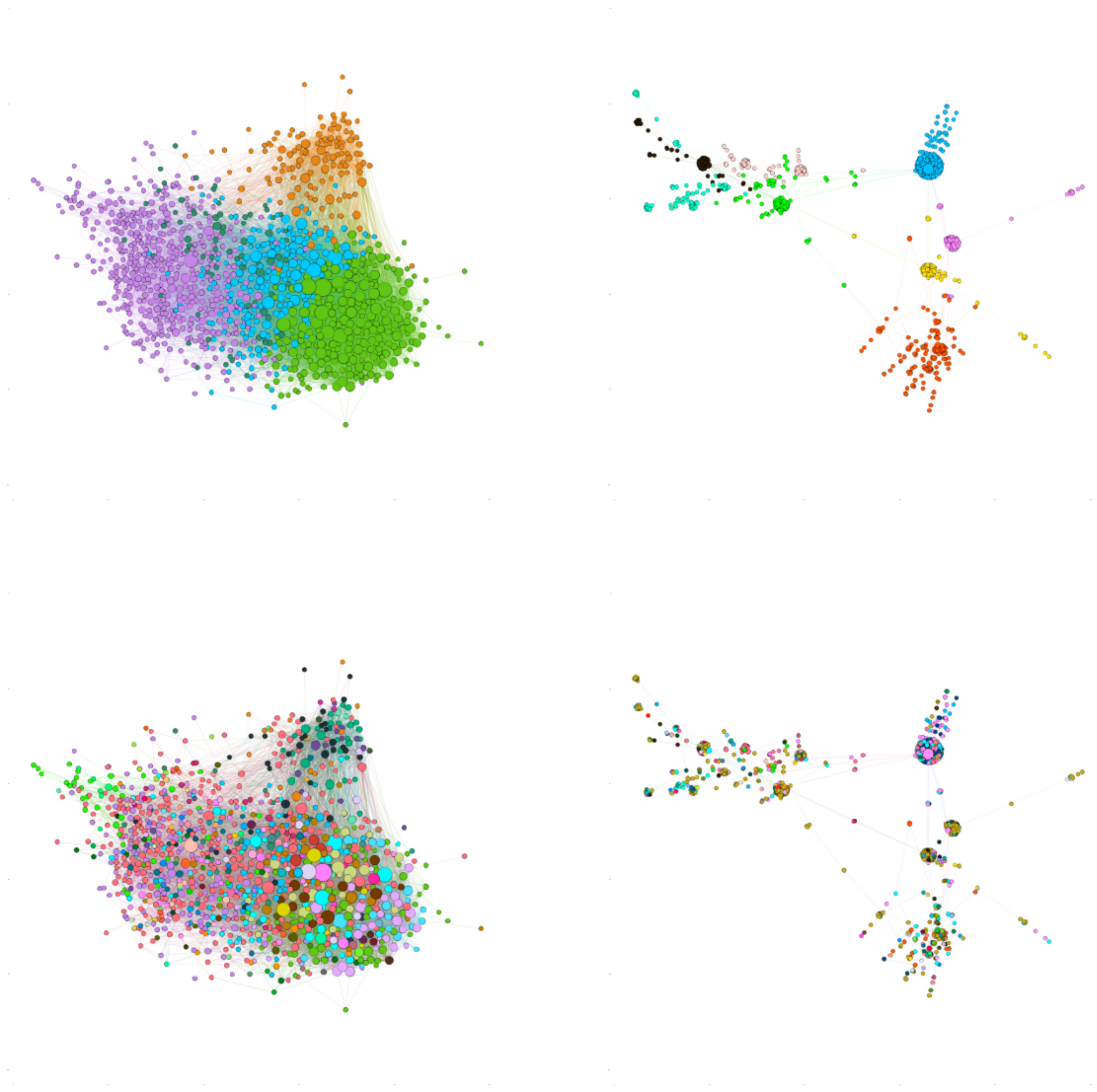

**Figure 7.** The retweet and friendship networks for the Twitter discussion about Depression. **Top left:** The friendship network color coded according the communities in the same friendship network. **Top right:** The retweet network color coded according to the communities in the same retweet network. **Bottom left:** The friendship network color coded according the communities in the corresponding retweet network. **Bottom right:** The retweet network color coded according to the communities in the corresponding friendship network. Excluding the isolated nodes, the retweet network for this discussion has 2655 nodes and 2727 edges, and the friendship network has 1113 nodes and 25589 edges. Small clusters are excluded from this visualization. The retweet networks visualized in this figure contain 1115 nodes and 1230 edges, and the friendship networks contain 1113 nodes and 25589 edges.

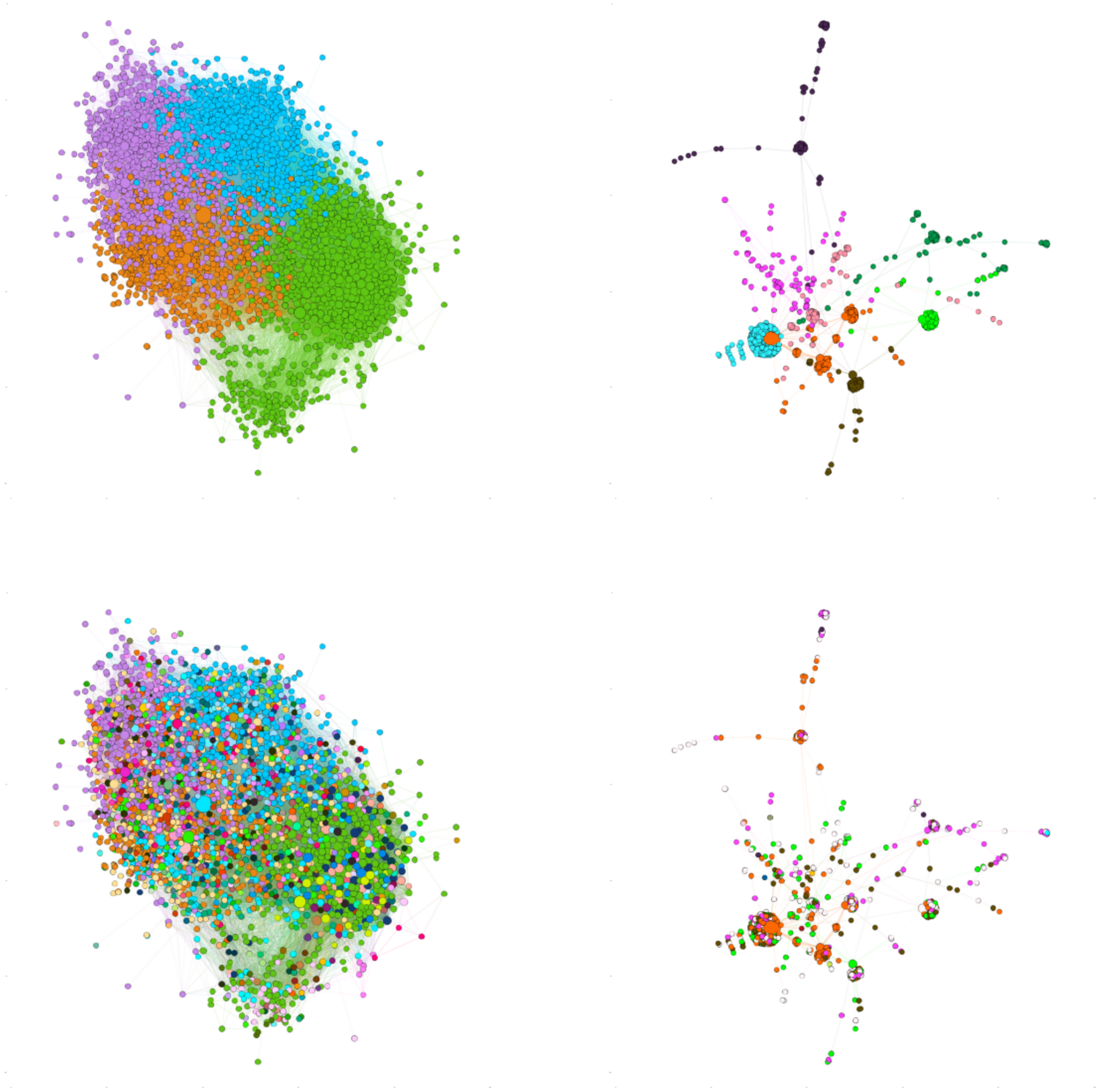

**Figure 8.** The retweet and friendship networks for the Twitter discussion about Golab Adineh. **Top left:** The friendship network color coded according the communities in the same friendship network. **Top right:** The retweet network color coded according to the communities in the same retweet network. **Bottom left:** The friendship network color coded according the communities in the corresponding retweet network. **Bottom right:** The retweet network color coded according to the communities in the corresponding friendship network. Excluding the isolated nodes, the retweet network for this discussion has 2118 nodes and 2192 edges, and the friendship network has 2676 nodes and 78339 edges. Small clusters are excluded from this visualization. The retweet networks visualized in this figure contain 910 nodes and 998 edges, and the friendship networks contain 2668 nodes and 78128 edges.

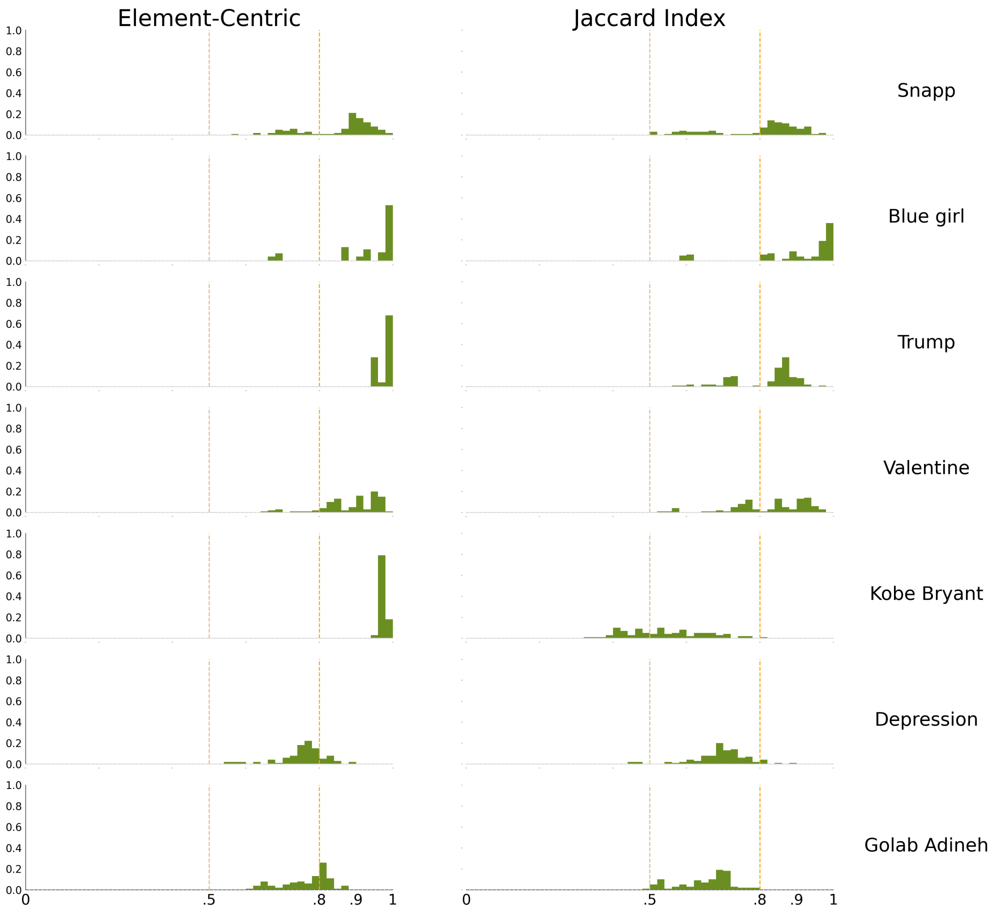


**Figure 9.** The distribution of similarity values between the Louvain clustering used in our analysis for the friendship networks and 100 alternative clusterings of the same networks obtained from 100 runs of the algorithms using different random seeds. On the left, the similarities are computed according to element-centric similarity. On the right, the similarities are computed according to Jaccard index.

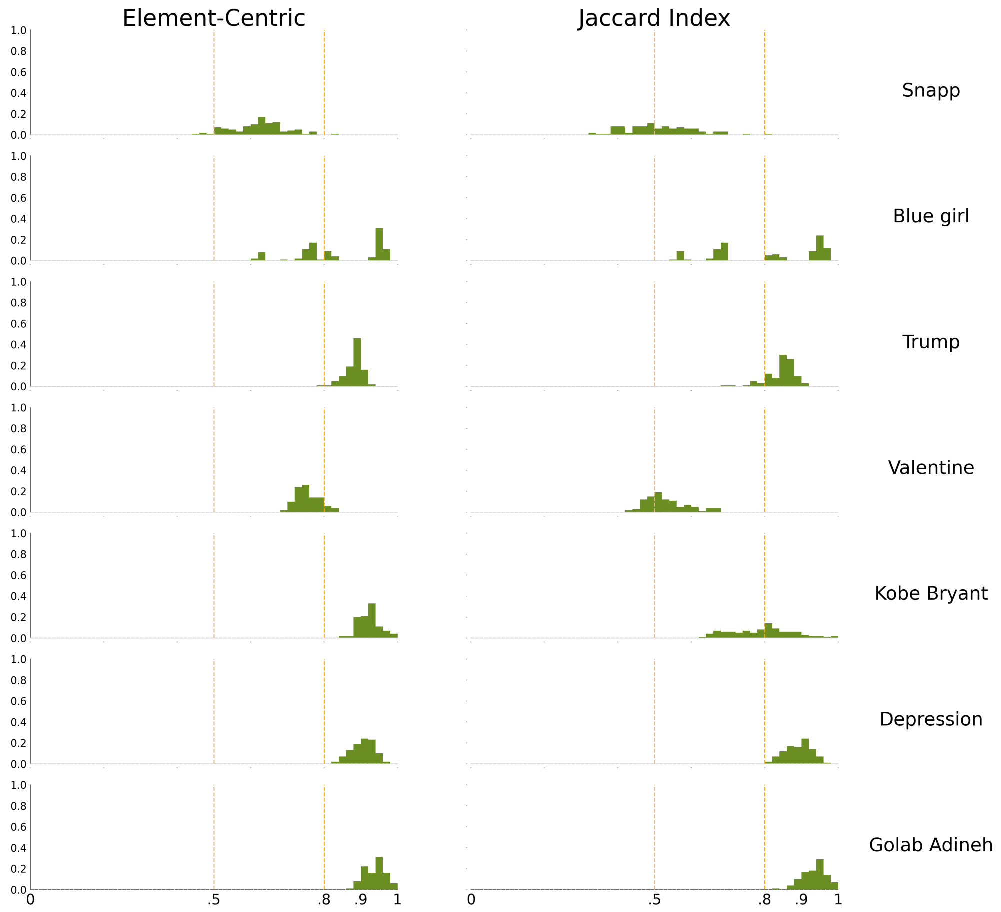


**Figure 10.** The distribution of similarity values between the Louvain clustering used in our analysis for the retweet networks and 100 alternative clusterings of the same networks obtained from 100 runs of the algorithms using different random seeds. On the left, the similarities are computed according to element-centric similarity. On the right, the similarities are computed according to Jaccard index.

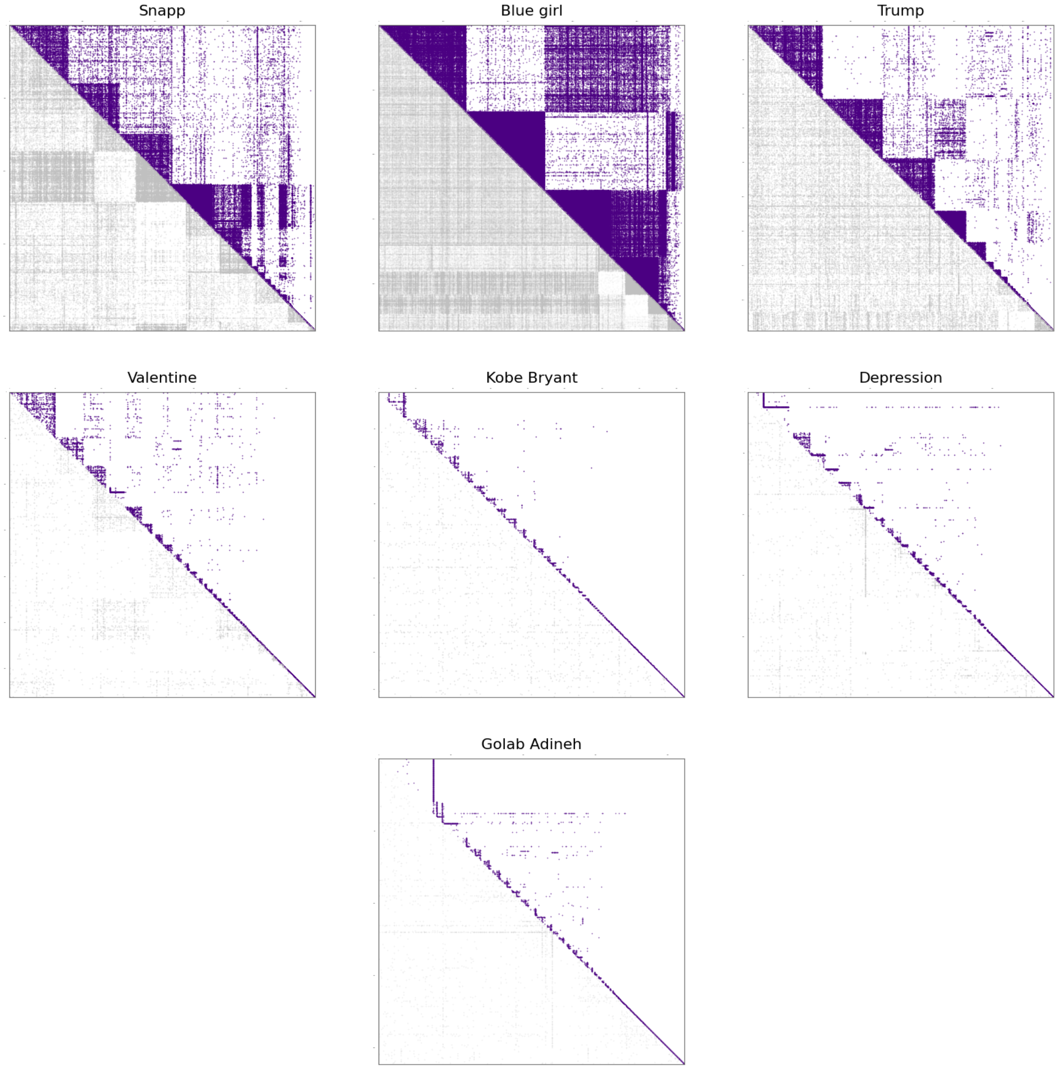


The symmetrized retweet networks for each of the divisive political (top row) apolitical (the rest) Twitter discussions. In the upper triangle the rows and columns are reordered such to group nodes by their retweet communities. In the lower triangle, the reordering groups nodes by the community they belong to in the corresponding friendship network. Excluding the isolated nodes, the retweet network of the Snapp discussion has 21029 nodes and 69565 edges, and the retweet network of the Valentine discussion has 3705 nodes and 4491 edges.

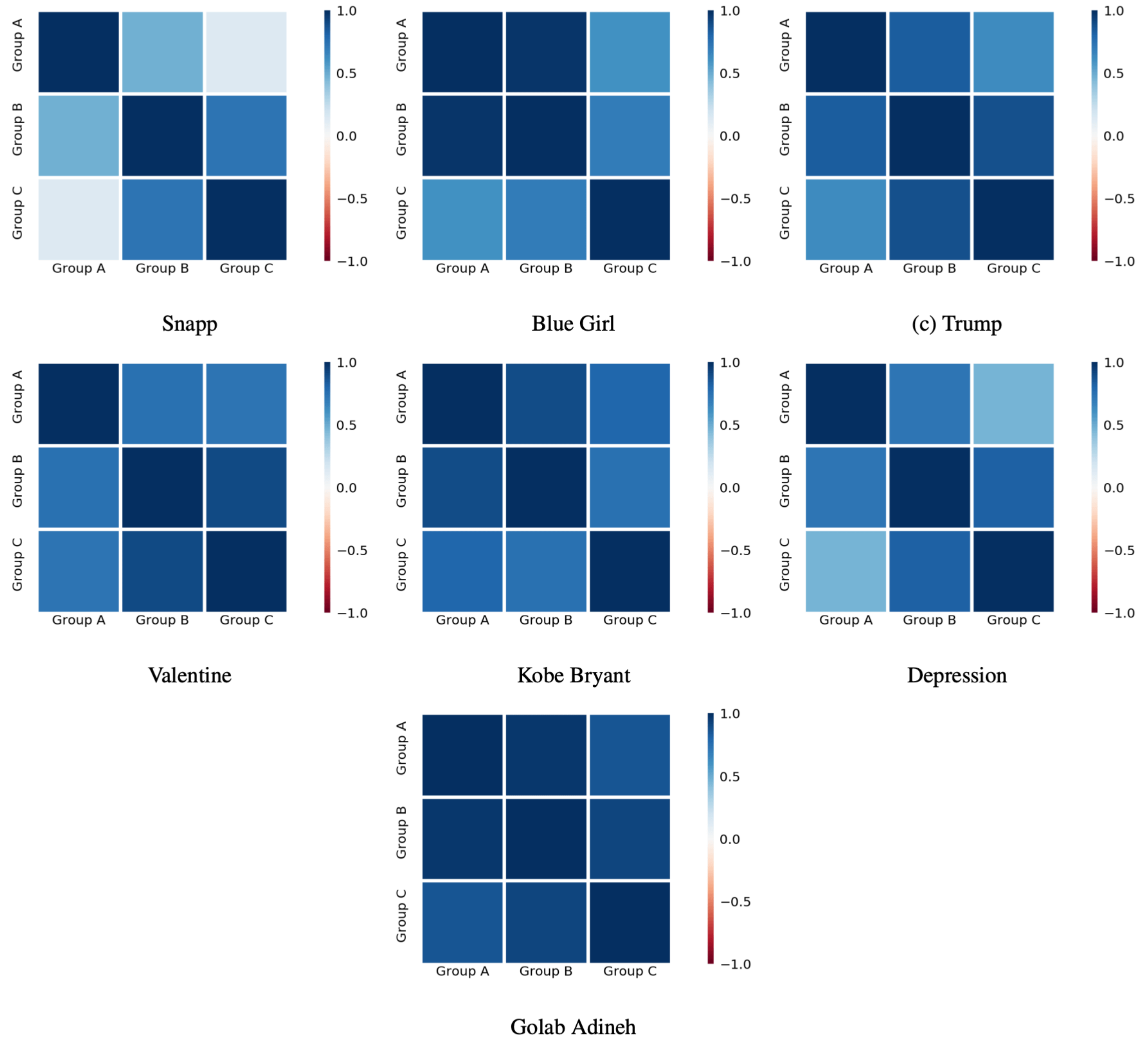


**Figure 12.** The Spearman correlation between the changes in the number of tweets from one day to the next for different groups of users — automated inauthentic accounts (group A), bot assisted humans and trolls (group B), and genuine users (group C). Please refer to Appendix section Topics of Discussion for descriptions of discussion topics and the number of tweets for each topic.

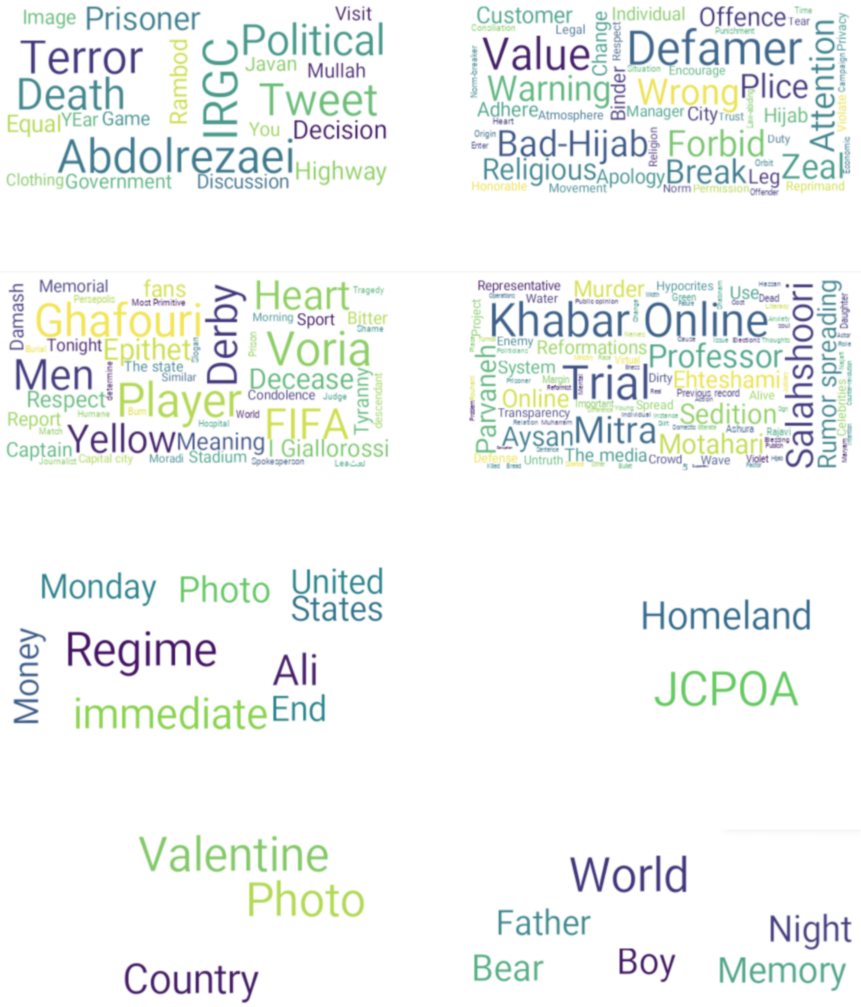


**Figure 13.** Difference word clouds by CAP for the Snapp, Blue Girl, Trump, and Valentine discussions, from top to bottom. Left: top 5% most frequent words used by users in major community 1 which are not among the top 10% most frequent words used by users in major community 2. Right: top 5% most frequent words used by users in major community 2 which are not among the top 10% most frequent words used by users in major community 1. Note that the difference word clouds for the apolitical discussions about Kobe Bryant, Depression, and Golab Adineh were empty, i.e. there is no difference between the two communities, in line with our main results.

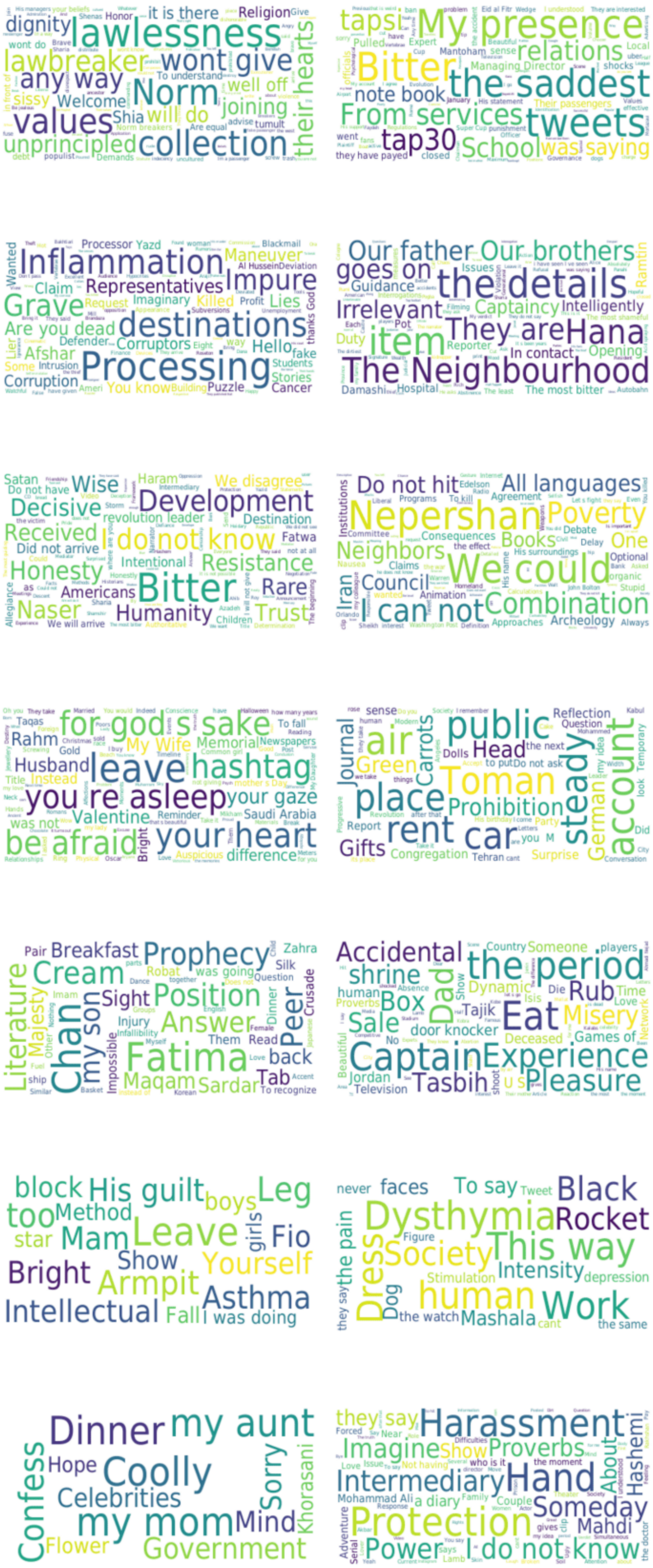


**Figure 14.** Difference word clouds by CAP for the Snapp, Blue Girl, Trump, Valentine, Kobe Bryant, Depression, and Golab Adineh discussions, from top to bottom. Left: top 5% most frequent words across the high-CAP group which are absent from the top 10% most frequent words used by the low-CAP group. Right: top 5% most frequent words across the low-CAP group which are absent from the top 10% most frequent words used by the high-CAP group.

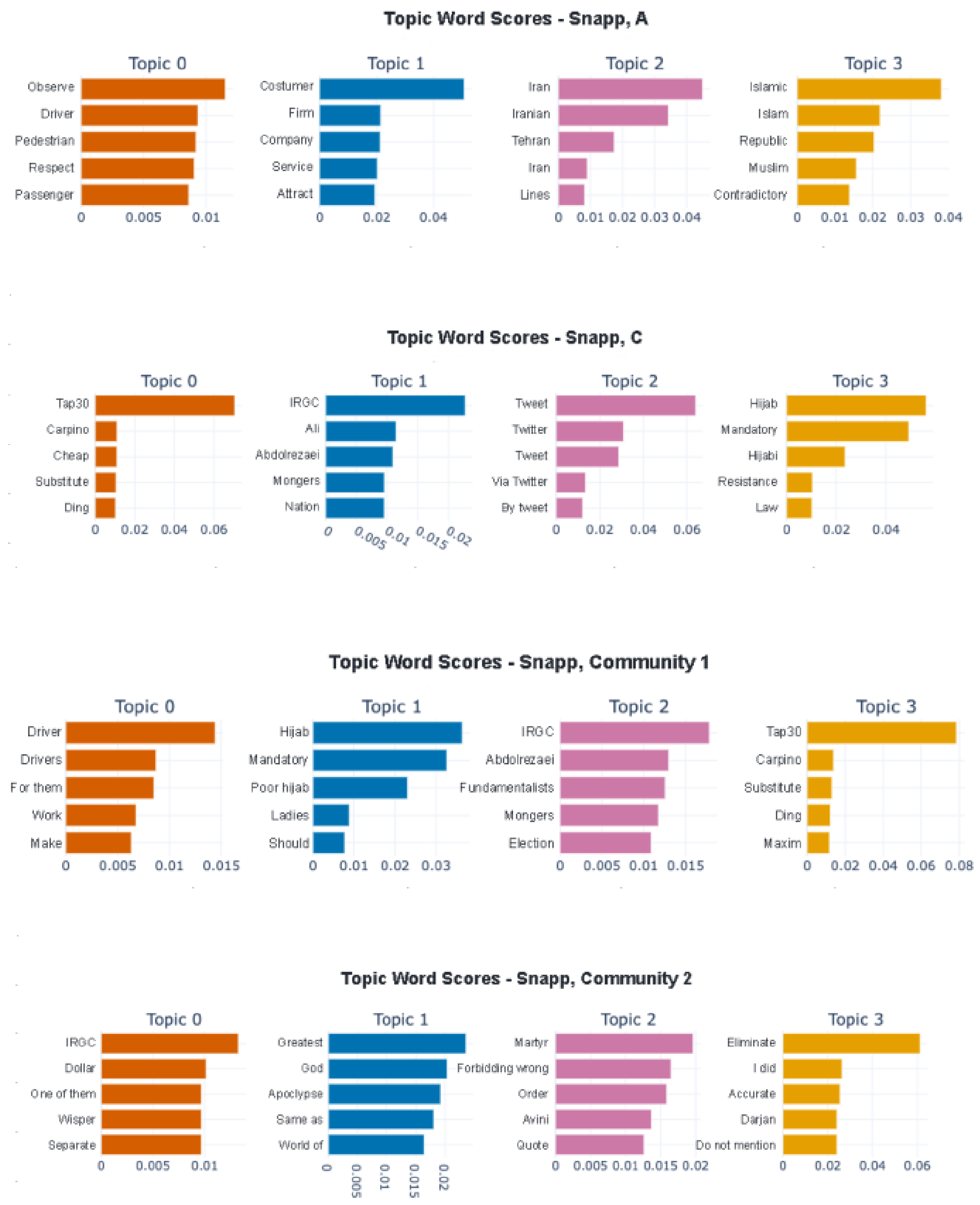


**Figure 15.** Salient topics and representative words, obtained from BERTopic, for tweets from low-CAP (C) and high-CAP (A) on the top two rows and from user in two major friendship communities on the bottom two rows, for the Snapp discussion.

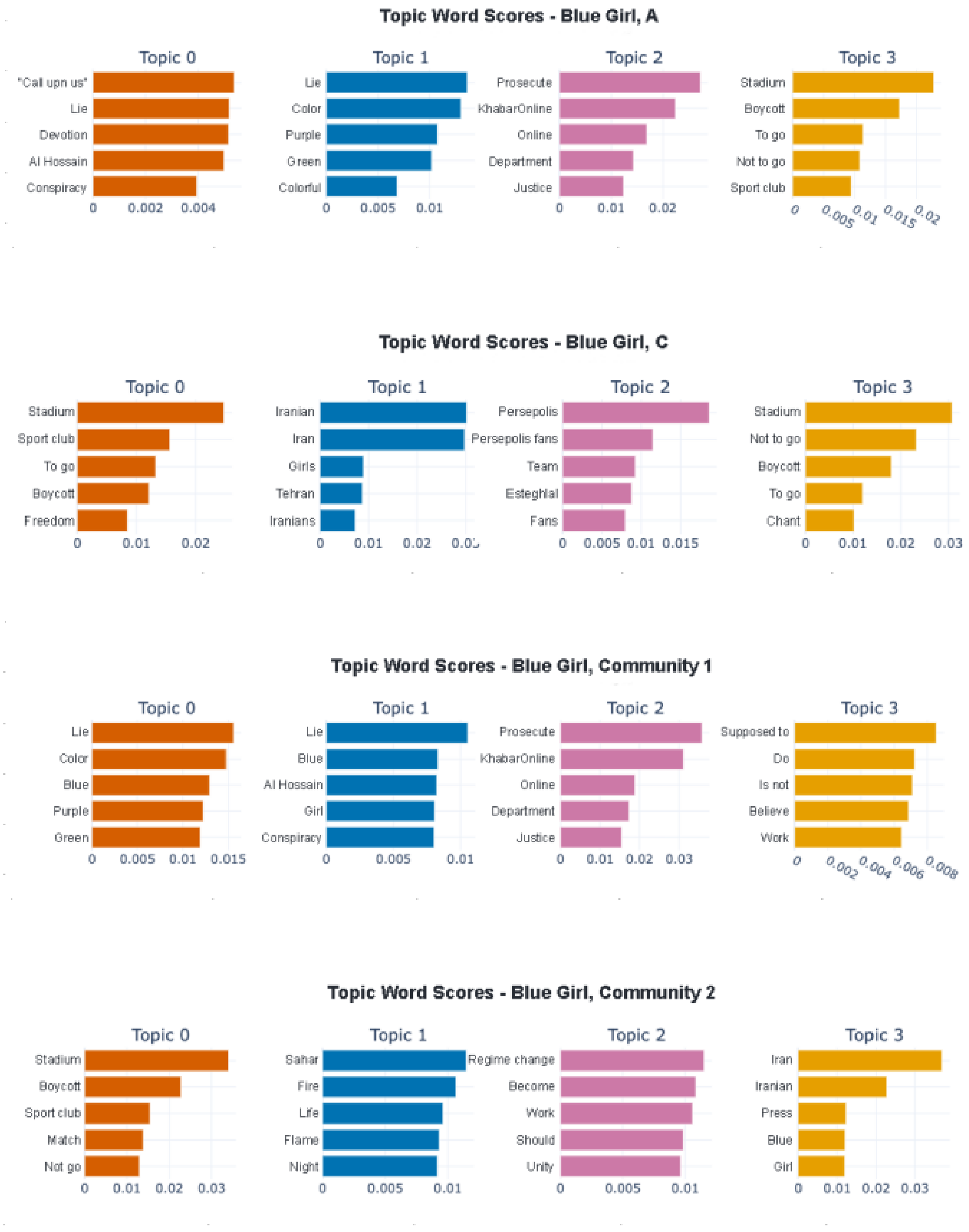


**Figure 16.** Salient topics and representative words, obtained from BERTopic, for tweets from low-CAP (C) and high-CAP (A) on the top two rows and from user in two major friendship communities on the bottom two rows, for the Blue Girl discussion.

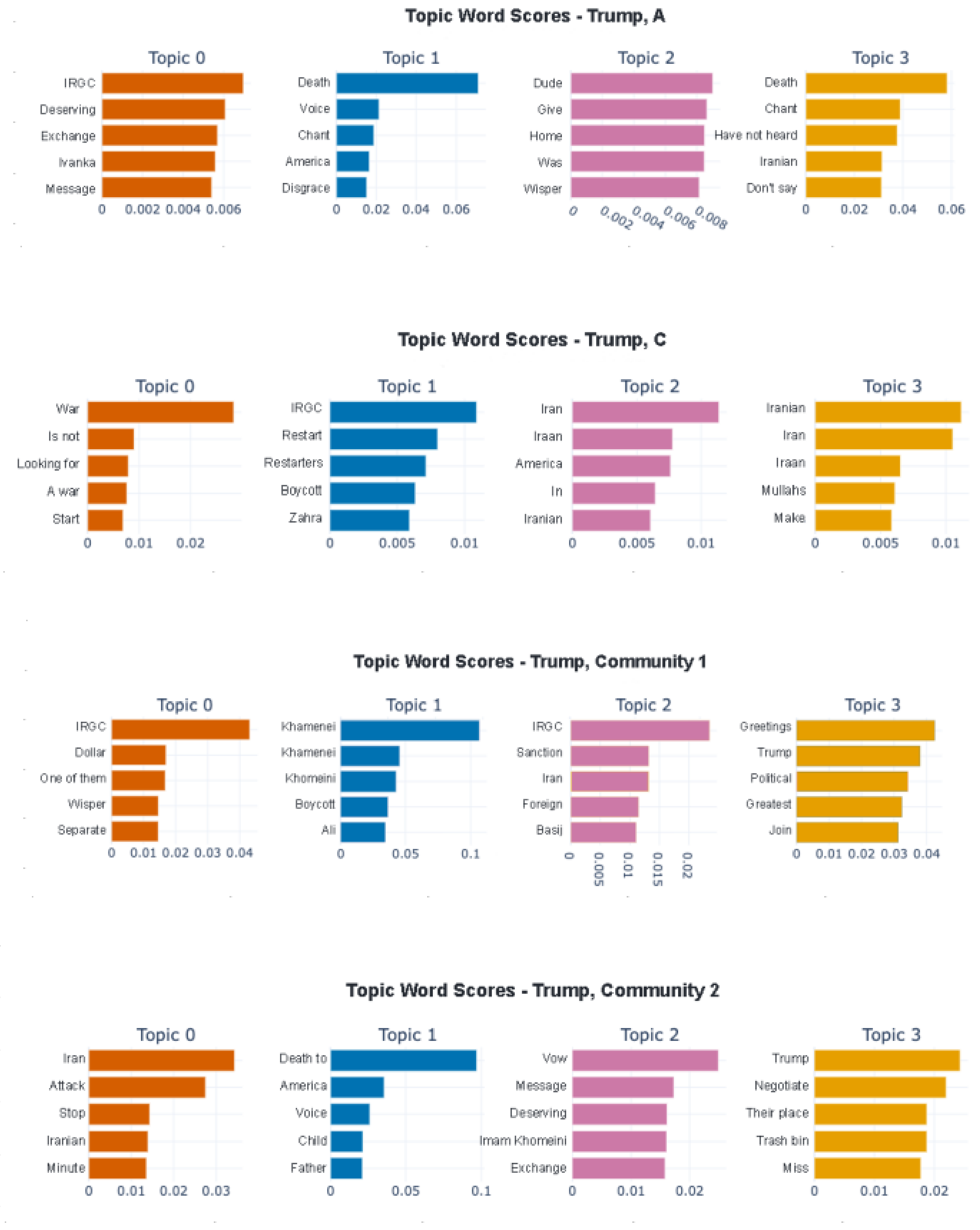


**Figure 17.** Salient topics and representative words, obtained from BERTopic, for tweets from low-CAP (C) and high-CAP (A) on the top two rows and from user in two major friendship communities on the bottom two rows, for the Trump discussion.

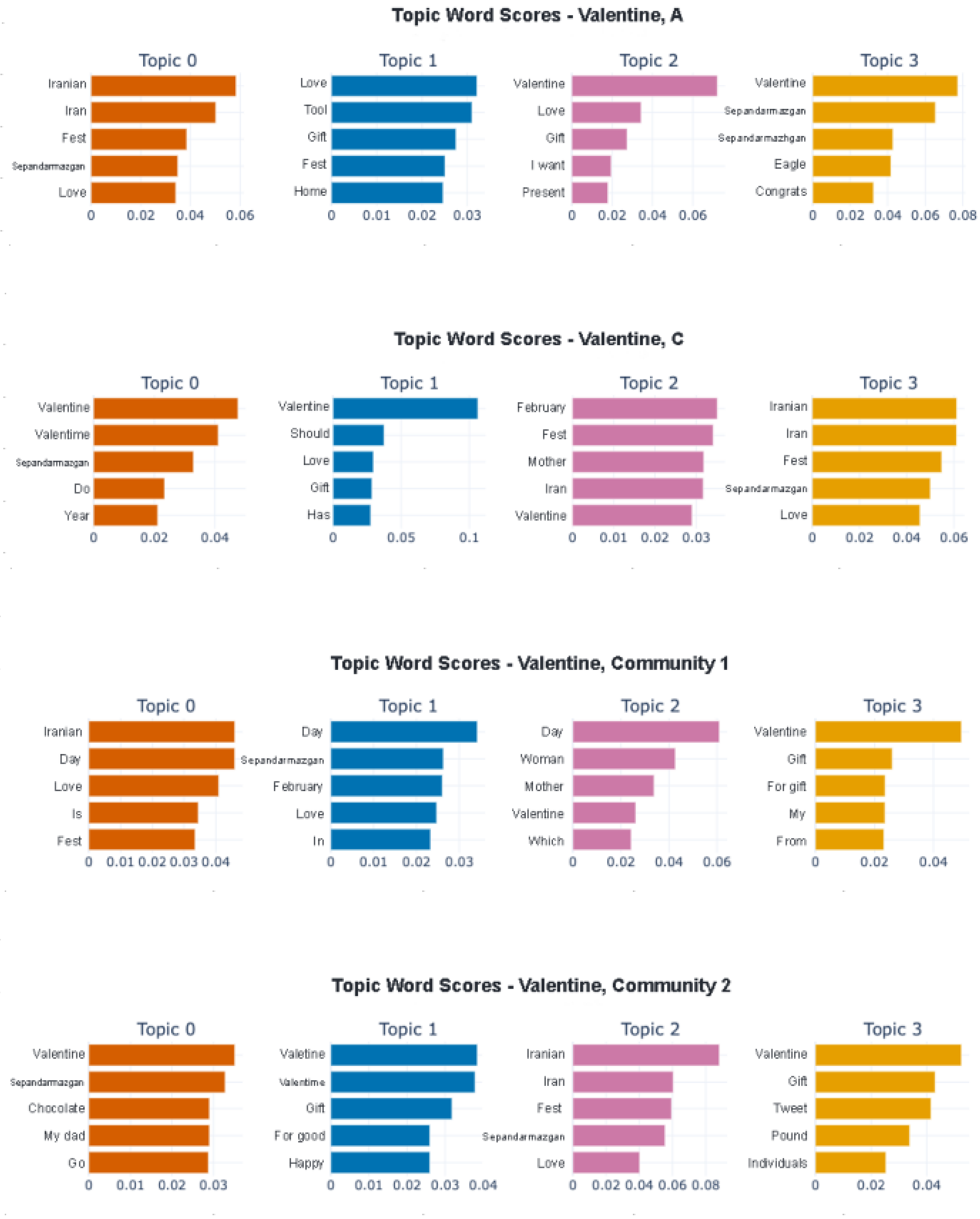


**Figure 18.** Salient topics and representative words, obtained from BERTopic, for tweets from low-CAP (C) and high-CAP (A) on the top two rows and from user in two major friendship communities on the bottom two rows, for the Valentine discussion.

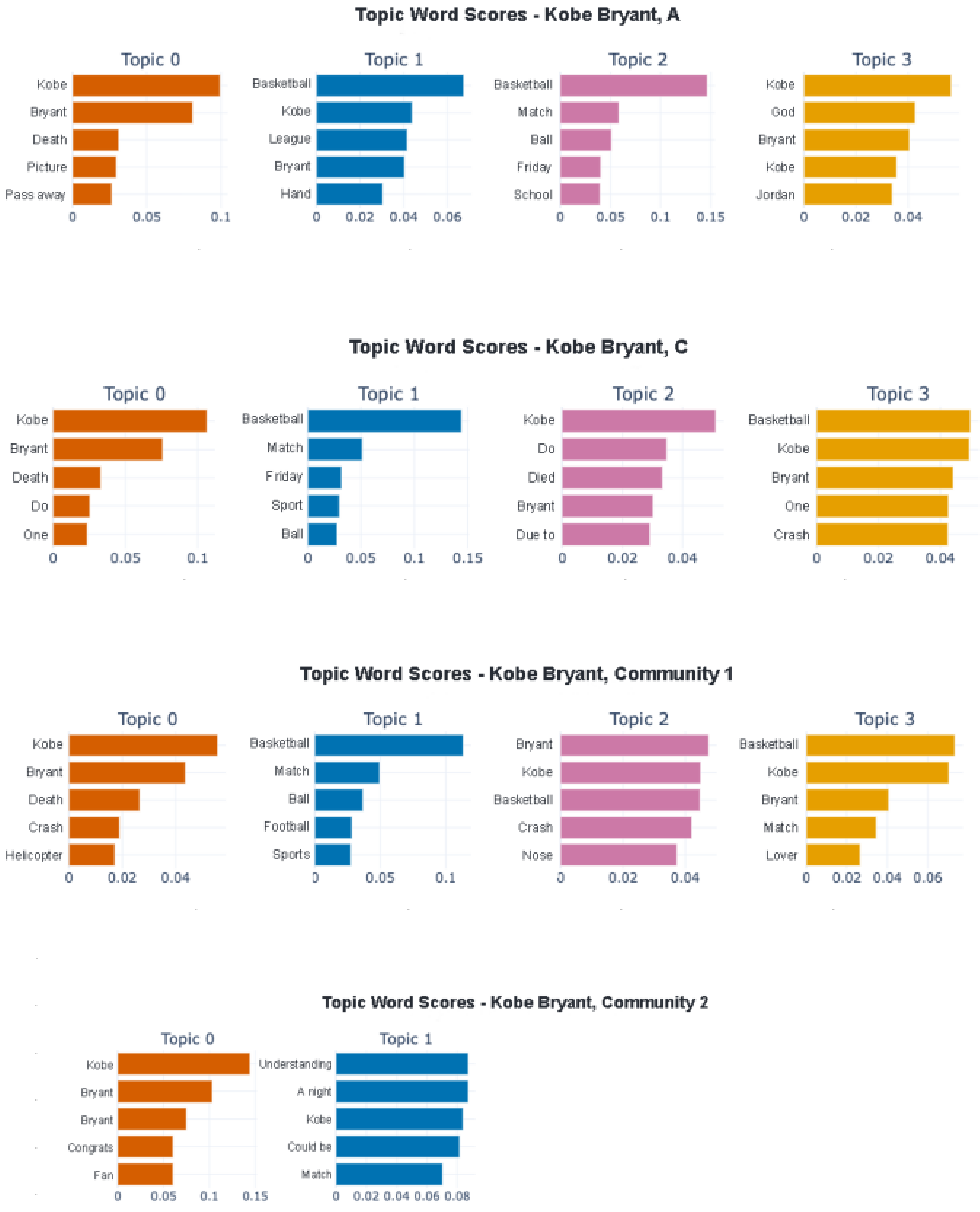


**Figure 19.** Salient topics and representative words, obtained from BERTopic, for tweets from low-CAP (C) and high-CAP (A) on the top two rows and from user in two major friendship communities on the bottom two rows, for the Kobe Bryant discussion.

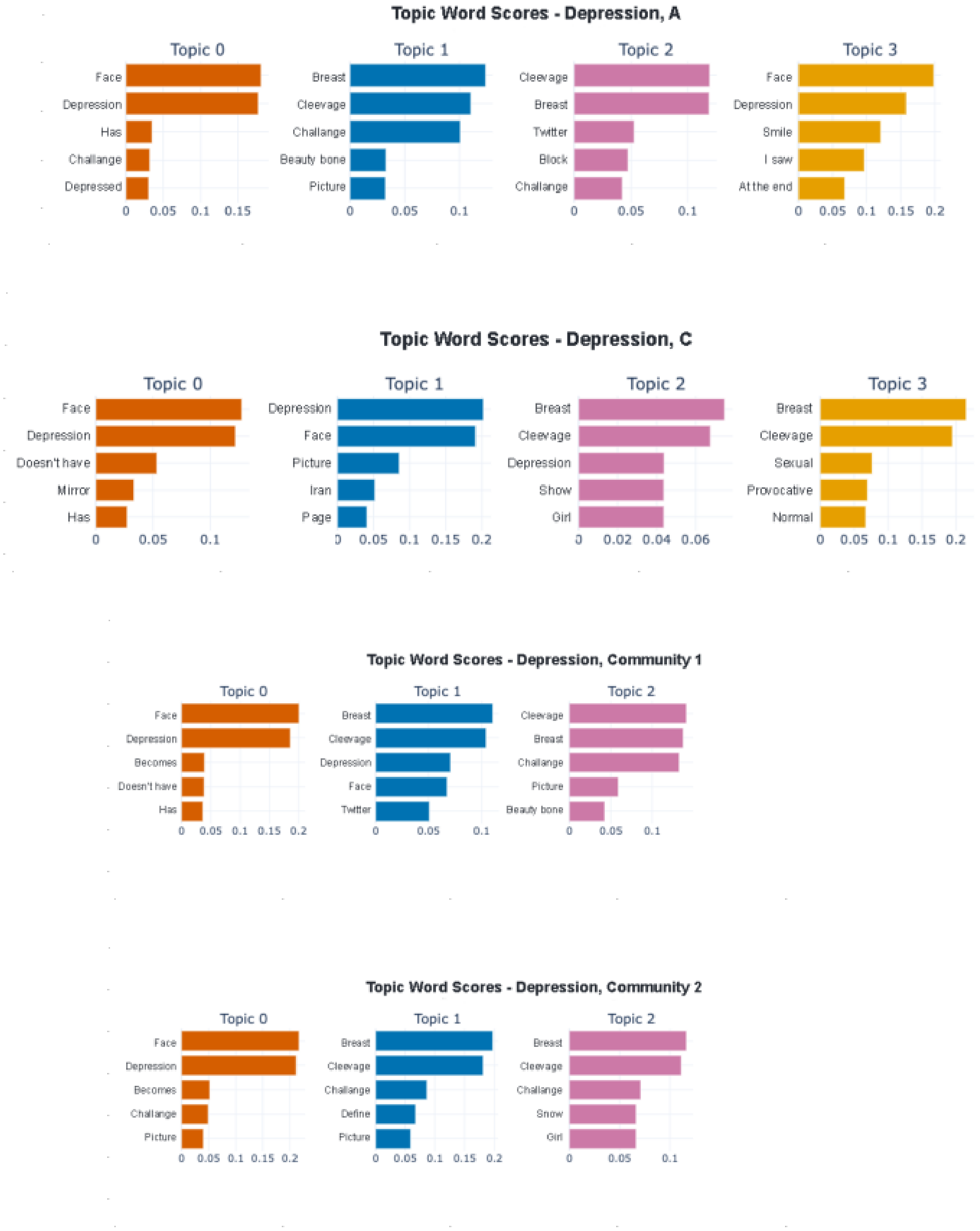


**Figure 20.** Salient topics and representative words, obtained from BERTopic, for tweets from low-CAP (C) and high-CAP (A) on the top two rows and from user in two major friendship communities on the bottom two rows, for the Depression discussion.

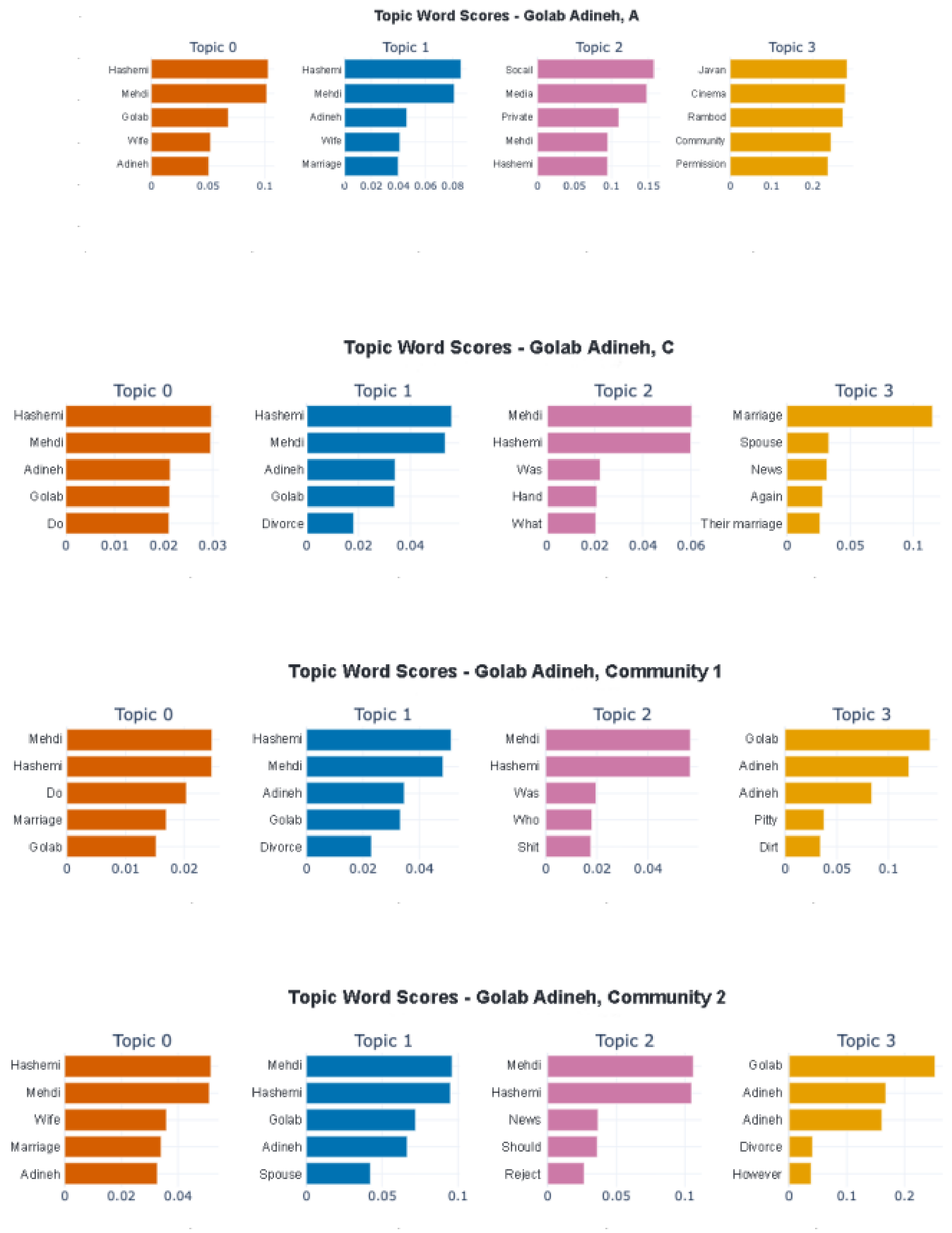


**Figure 21.** Salient topics and representative words, obtained from BERTopic, for tweets from low-CAP (C) and high-CAP (A) on the top two rows and from user in two major friendship communities on the bottom two rows, for the Golab Adineh discussion.
